# Phylogenetic analysis of avian schistosome *Trichobilharzia regenti* (Schistosomatidae, Digenea) from naturally infected hosts in northern Iran

**DOI:** 10.1002/vms3.1225

**Published:** 2023-07-25

**Authors:** Elham Kia Lashaki, Arezoo Bozorgomid, Shirzad Gholami, Mehdi Karamian, Mahdi Fakhar, Samira Dodangeh

**Affiliations:** ^1^ Department of Parasitology and Mycology School of Medicine, Tonekabon Branch, Islamic Azad University Tonekabon Iran; ^2^ Infectious Diseases Research Center Health Institute Kermanshah University of Medical Sciences Kermanshah Iran; ^3^ Toxoplasmosis Research Center Iranian National Registry Center for Lophomoniasis and Toxoplasmosis School of Medicine, Mazandaran University of Medical Sciences Sari Iran; ^4^ Department of Parasitology and Mycology School of Medicine, Zanjan University of Medical Sciences Zanjan Iran; ^5^ Children Growth Research Center Research Institute for Prevention of Non‐Communicable Diseases, Qazvin University of Medical Sciences Qazvin Iran

**Keywords:** aquatic birds, aquatic snails, COX1, Iran, ITS1, phylogenetic analysis, *Trichobilharzia regenti*

## Abstract

**Background:**

*Trichobilharzia regenti* (*T. regenti*) is an avian schistosomatid fluke species that causes human cercarial dermatitis (HCD) in areas of aquaculture in northern Iran. Understanding the phylogenetic relationships and genetic diversity of this thread‐like fluke will deepen our thoughtful of avian schistosomiasis epidemiology and lead to more effective HCD control in the region.

**Objectives:**

To determine the life cycle of nasal Trichobilharzia in aquatic birds as well as aquatic snails and also identify the haplotype diversity of the isolates in Mazandaran Province, northern Iran.

**Methods:**

In the present study, adult or egg of *Trichobilharzia* isolated from aquatic birds as well as schistosomes cercariae isolated from aquatic snails in Mazandaran Province, northern Iran, belonged to the authors' previous research, were examined. Molecular studies and phylogenetic analysis were carried out on these schistosomes samples.

**Results:**

The phylogenetic analysis of the ITS1 and COX1 genes in isolated schistosomes revealed that all samples belong to the *T. regenti* clade. Remarkably, based on phylogenetic results, these schistosomes samples from *Anas platyrhynchos domesticus*, *A. platyrhynchos, Spatula clypeata* and *Lymnaea stagnalis* grouped together with previously sequenced samples from Iran (*Trichobilharzia cf. regenti*). Unlike the phylogenetic tree and haplotype network of COX1 gene, ITS1 did not show distinct clusters.

**Conclusion:**

This study completed the puzzle of the disease in Mazandaran Province by isolating and genotyping furkocercariae from *L. stagnalis* that was consistent with the isolated new genotype from ducks. For the first time in Iran, this confirmed the potential role of *L. stagnalis* snails in the transmission of the disease.

## Graphical abstract

This article demonstrates new information about the current status of *Trichobilharzia* species in infected ducks and snails in the aquaculture areas of Mazandaran Province in northern Iran. The new genotype of *Trichobilharzia cf. regenti* is circulating between migratory and domestic ducks in the study areas. Furthermore, by isolating and genotyping furkocercariae from *L. stagnalis* and finding consistency with isolated new genotypes from ducks, this study completed the puzzle of the disease in Mazandaran Province for the first time in Iran.

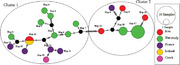

## 1 INTRODUCTION

Transmission of the avian schistosomes (Digenea: Schistosomatidae) to humans occurs when the free‐swimming larval form (cercaria) emerges from the intermediate hosts and accidentally penetrates human skin. These cercariae do not mature and typically die in the skin, causing human cercarial dermatitis (HCD), also known as swimmer's itch. HCD is considered an emerging and reemerging infectious disease in various parts of the World (Horák et al., 2015). The global prevalence of avian schistosomes is estimated at 34.0% with *Allobilharzia visceralis* and *Trichobilharzia* spp. being the most commonly reported in birds (Lashaki et al., 2020). The genus *Trichobilharzia* is one of the most common types of avian schistosomes found extensively on all continents except Antarctica (Horák et al., 2015). Within this genus, there are 40 species that infect five orders of waterfowl as definitive hosts, while four families of freshwater gastropods serve as intermediate hosts (Brant & Loker, 2009). Adult worms of *Trichobilharzia* live in the mesenteric or nasal veins of their definitive hosts (Jouet et al., 2009).

*Trichobilharzia regenti* is one of four common species found in Europe. The cercariae of *T. regenti*, similar to visceral schistosomes, develop into schistomula in the host skin. However, before settling in the nasal cavity of the definitive avian host, the immature flukes travel through the peripheral nerves to the brain (Horák et al., 1999; Hrádková & Horák, 2002). This migration through the central nervous system (CNS) allows the parasites to feed on the host's nervous tissue. As a result, the characteristics of *T. regenti* are significant due to the potential pathological risks inherent in this migration route (Lichtenbergová et al., 2011). *T. regenti* has been identified as the causative agent of HCD in Europe and North America (Jouet et al., 2009). The most common intermediate host snails for *T. regenti* in Europe (and possibly in Iran) are *Radix balthica* ( *= R. peregra*) and *R. gedrosiana* (also known as *Lymnaea gedrosiana*) (Imani‐Baran et al., 2012; Rudolfová et al., 2007).

HCD is considered an occupational health problem in certain regions of Iran (Gholami et al., 2021). In northern Iran, paddy farmers are particularly prone to experiencing exanthema on their legs and arms as a result of contact with waters that contain snails infected with schistosome larvae (Gholami et al., 2021). To better understand the causes of HCD in Iran, multiple studies have been conducted on the final host birds and intermediate host snails (Gholami et al., 2021; Gohardehi et al., 2013; Maleki et al., 2012; Yakhchali et al., 2016).

In a previous study conducted by the authors of this article on nasal avian schistosomes in northern Iran (Fakhar et al., 2016), the genetic diversity of these parasites was analysed by sequencing the ITS1 and COX1 fragments obtained from parasites isolated from waterfowl. In that study, the researchers identified a specific *T. regenti*‐like haplotype from the COX1 *Trichobilharzia* fragment, which had previously only been found in the anatidae family in studies conducted in France and Poland, and whose snail host had not yet been identified (Jouet et al., 2009; Jouet et al., 2010).

In the current study, we examined samples of *Trichobilharzia* eggs collected from the nasal region of birds as well as schistosome cercariae isolated from aquatic snails in the same geographic areas to identify the desired haplotype and determine the life cycle of nasal *Trichobilharzia* in birds in this region.

**Impacts**

- This article shows new information on the present status of *Trichobilharzia* spp., in infected ducks and snails in aquaculture areas in Mazandaran Province, northern Iran.
- Our report reveals that a new genotype of *Trichobilharzia* is circulating in the study areas among both migratory and domestic ducks. This finding confirms that the establishment of the disease cycle is not solely dependent on migratory birds.
- Our data demonstrate the potential role of *Lymnaea stagnalis* in transmitting the disease.

## 2 MATERIALS AND METHODS

### 2.1 Ethics approval

The current study was reviewed and approved by the Ethical Committee of Mazandaran University of Medical Sciences, Sari, Iran (IR.MAZ.REC.1397.1692).

### 2.2 Sample collection

The study was conducted in Mazandaran Province, northern Iran. This province is located in the eastern part of the southern coast of the Caspian Sea and bordered clockwise by Russia (across the sea) and the provinces of Golestan, Semnan, Tehran, Alborz, Qazvin and Guilan. It has a humid and subtropical climate with an average temperature of 17°C. We analysed adult *Trichobilharzia* and *Trichobilharzia* eggs from aquatic birds, as well as schistosoma cercariae taken from aquatic snails that were obtained in previous research. The positive samples examined in this study included 23 migrating ducks belonging to *Anas platyrhynchos* and *Spatula clypeata*, 16 domestic ducks belonging to *Anas platyrhynchos domesticus* and *Aythya ferina*, and three freshwater snails belonging to *Lymnaea stagnalis* and *Radix auricularia*. In our earlier study, 41 out of 255 birds tested for nasal schistosomes were found to be positive (an infection rate of 16%), and 0.17% of snails collected from the same area were infected with avian schistosomes.

### 2.3 DNA extraction and PCR amplification

Identification of avian schistosomes eggs and ocellate furcocercariae was previously performed using light microscopy. The isolates were stored in ethanol and transferred to the Helminthology Laboratory of Mazandaran University of Medical Sciences, Sari, Iran for molecular studies. Cercariae obtained from the isolates were pooled into 1.5 mL microfuge tubes (30–40 cercariae per snail). Genomic DNA was manually extracted from furcocercariae with the modified salting out method as previously described (Gholami et al., 2021). For *Trichobilharzia* eggs, DNA was extracted using a commercial kit (DENAzist Asia Animal Tissue DNA Isolation Kit) according to the manufacture's protocol. To amplify the internal transcribed spacer 1 (ITS1) region of the nuclear rDNA, we used primers BD1 (5′‐GTCGTAACAAGGTTTCCGT‐3′) and 4S (50‐ACCACTAACTAATTCACTTTC‐30). The mitochondrial DNA cytochrome oxidase 1 (COX1) region was amplified using the primers Cox1_Schisto F (5′‐TCTTTRGATCATAAGCG‐3′) and Cox1_Schisto R (5′‐TAATGCATMGGAAAAAAACA3′). The PCR reactions were performed in a total volume of 25 μL containing 200 μM of deoxynucleotide triphosphates (dNTPs), 2.5 μL of 10X PCR buffer, 0.7 mg/μL MgCl_2_, 0.6 units of Taq polymerase, 10 pmol of each primer and 2 μL of sample DNA. The PCR cycle was performed in a Thermal Cycler (Eppendorf) with an initial 3 min denaturation at 94°C, followed by 30 amplification cycles (denaturation at 94°C for 45 s, annealing at 54°C and 56°C for 45 s for ITS1 and COX1, respectively, and elongation at 68°C for 2 min followed by a final extension step at 68°C for 10 min. Afterwards, the PCR products were analysed using 1.5% agarose gel electrophoresis containing SimplyBlue Safe stain alongside a 100 bp DNA marker (SinaClon). Finally, 23 samples of PCR products were sequenced in both directions with the same primers used in the PCR reaction (Bioneer Company).

### 2.4 Phylogenetic analysis

Phylogenetic analyses were performed using both newly obtained sequences, and ones downloaded from GenBank (Tables 1, 2, 3). Forward and reverse sequences were assembled using ChromasPro version 1.7.5 (Technelysium). The sequences obtained in this study were manually trimmed and edited alongside previously published ITS1 and COX1 sequences from *T. regenti* isolates in BioEdit v.7.2 software. Ambiguous (heterozygous) sites were coded using the standard IUPAC codes for combinations of two or more bases. The maximum‐likelihood phylogram (ML) was constructed in MEGA (version 6.0; Biodesign Institute) using HKY+G (Hasegawa‐Kishino‐Yano) and TN93+G (Tamura‐Nei, 93) for ITS1 and COX1 regions, respectively, which were chosen as the most appropriate substitution model. Node support was assessed with 1000 bootstrap replicates.

**TABLE 1:** Isolates of bird schistosomes used for molecular analysis in the present study.

Isolate	Stage	Host	Origin	Coordinates (latitude/longitude)	Accession numbers		Haplotype no	
					ITS1	COI	ITS1	COI
SC1	Egg	*Anas clypeata*	Ezbaran	36.6505° N, 52.4905° E		MN337548		3
SC3	Egg	*Anas clypeata*	Seyed Mahale	36.4724° N, 52.8650° E		MN337549		17
AP18	Egg	*Anas platyrhynchos*	Mahmudabad	36.6329° N, 52.2667° E		MN337554		18
APD10	Egg	*Anas platyrhynchos domesticus*	Seyed Mahale	36.4724° N, 52.8650° E	MN337567	MN337558	2	17
APD13	Egg	*Anas platyrhynchos domesticus*	Mahmudabad	36.6329° N, 52.2667° E		MN337559		4
SC8	Egg	*Anas clypeata*	Sorkhrud	36.6717° N, 52.4439° E		MN337551		17
APD3	Egg	*Anas platyrhynchos domesticus*	Ezbaran	36.6505° N, 52.4905° E	MN337565	MN337556	2	6
SC6	Egg	*Anas clypeata*	Mahmudabad	36.6329° N, 52.2667° E		MN337550		7
SC11	Egg	*Anas clypeata*	Sorkhrud	36.6717° N, 52.4439° E		MN337552		18
AP16	Egg	*Anas platyrhynchos*	Ezbaran	36.6505° N, 52.4905° E	MN337563	MN337553	5	5
AP21	Egg	*Anas platyrhynchos*	Ezbaran	36.6505° N, 52.4905° E	MN337564	MN337555	4	6
APD5	Egg	*Anas platyrhynchos domesticus*	Ezbaran	36.6505° N, 52.4905° E	MN337566	MN337557	3	5
RA2	Cercaria	*Radix auricularia*	Ezbaran	36.6505° N, 52.4905° E	MN337568	MN337560	1	16
RA3	Cercaria	*Radix auricularia*	Ezbaran	36.6505° N, 52.4905° E	MN337569	MN337561	1	9
LS1	Cercaria	*Lymnaea stagnalis*	Ezbaran	36.6505° N, 52.4905° E	MN337570	MN337562	1	10

**TABLE 2:** GenBank sequences of bird schistosomes used for the mitochondrial COX1 phylogenetic analysis and the reference.

Isolate	Host	Locality	Accession number	Author	Year
*T. regenti*	*Mergus merganser*	France	HM439501	Jouet et al.	2010
*T. regenti*	*Anas platyrhynchos*	Iceland	HM439503	Jouet et al.	2010
*T. regenti*	*Cygnus olor*	France	HM439500	Jouet et al.	2010
*T. regenti*	*Radix peregra*	France	HM439499	Jouet et al.	2010
*T. regenti*	*Anas platyrhynchos*	France	HM439502	Jouet et al.	2010
*T. regenti*	*Radix peregra*	Czech Republic	AY157190	Lockyer et al.	2003
*Trichobilharzia sp. JIT11*	*Spatula clypeata*	France	HM439505	Jouet et al.	2010
*Trichobilharzia cf. regenti*	*Anas platyrhynchos*	Iran	KR108326	Fakhar et al.	2016
*Trichobilharzia cf. regenti*	*Spatula clypeata*	Iran	KR108325	Fakhar et al.	2016
*Trichobilharzia anseri*	*Anser anser*	France	KP901385	Jouet et al.	2015
*Schistosoma bovis*	*Mus musculus*	Tanzania	AY157212	Lockyer et al.	2003

**TABLE 3:** GenBank sequences of bird schistosomes used for the mitochondrial ITS1 phylogenetic analysis and the reference.

Isolate	Host	Locality	Accession number	Author	Year
*Trichobilhara regenti*	*Anas platyrhynchos*	Iran	MH410290	Ashrafi et al.	2018
*Trichobilhara regenti*	*Spatula clypeata*	Czech Republic	EF094540	Rudolfova et al.	2006
*Trichobilhara regenti*	*Radix peregra*	Denmark	KP271015	Christiansen	2016
*Trichobilhara regenti*	*Ampullaceana balthica*	Denmark	MW482442	Al‐Jubury et al.	2021
*Trichobilhara regenti*	*Anas platyrhynchos*	Poland	EF094535	Rudolfova et al.	2006
*Trichobilharzia regenti*	*Ampullaceana balthica*	Denmark	MW482438	Al‐Jubury et al.	2021
*Trichobilharzia regenti*	*Radix peregra*	Czech Republic	AF263829	Dvorak et al.	2002
*Schistosoma japonicum*	*Oncomelania hupensis hupensis*	China	FJ852552	Zhao et al.	2012

### 2.5 Genetic differentiation and haplotype network analysis

Molecular variation was characterised by several parameters, including the number of haplotypes, haplotype diversity (h), nucleotide diversity (p), number of segregating sites and indices such as Tajima's D and Fu's Fs statistic, which were estimated using DnaSP software (version 5.10). Additionally, the haplotype network inferred based on haplotypes of ITS1 and COX1 regions was constructed by PopART software using TCS algorithm.

## 3 RESULTS

### 3.1 Nucleotide sequence analysis

The PCR products were sequenced directly using the primers for 1ITS and COX1 fragments, which were used for DNA amplification. The sequences have been deposited in GenBank and can be accessed via the accession numbers MN337548 to MN337570. To ensure accuracy, these sequences were compared with existing records in GenBank.

### 3.2 Variations in nucleotide sequences

The COX1 (1244 bp) and ITS1 (1421 bp) genes were successfully amplified from 15 and 8 isolates, respectively. After trimming the sequences, fragment lengths of 999 and 1421 bp were obtained for COX1 and ITS1, respectively, and were used for subsequent analysis. Thirty‐seven variable sites were observed in the COX1 sequence, resulting in the identification of 10 haplotypes. The total haplotype diversity and nucleotide diversity (per site) were found to be 0.895 ± 0.070 and 0.0152 ± 0.001, respectively (as shown in Table 4). Similarly, for the ITS1 gene, 10 variable sites were found, resulting in the identification of five haplotypes. The total haplotype diversity and nucleotide diversity (per site) were found to be 0.857 ± 0.108 and 0.00213 ± 0.00073, respectively (as shown in Table 4).

**TABLE 4:** Details of schistosoma samples detected in the study, haplotypes (COX1 and ITS1) and Genbank accession numbers of the corresponding newly generated sequences.

Locus	Length (bp)	Sample size	S	No. Ha	Hd	Pi	Tajima's D	Fu's Fs
COX1	999	15	37	10	0.8952	0.01523	1.42824^a^	1.195^a^
ITS1	1421	8	10	5	0.857	0.00213	−1.37475^a^	−0.44^a^

S, number of polymorphic sites; No. Ha, number of haplotypes; π Hd, haplotype diversity; Pi, nucleotide diversity.

^a^ *p* > 0.05.

### 3.3 Results of sequencing of COX1 gene region, phylogenetic tree and haplotype network

**FIGURE 1:**
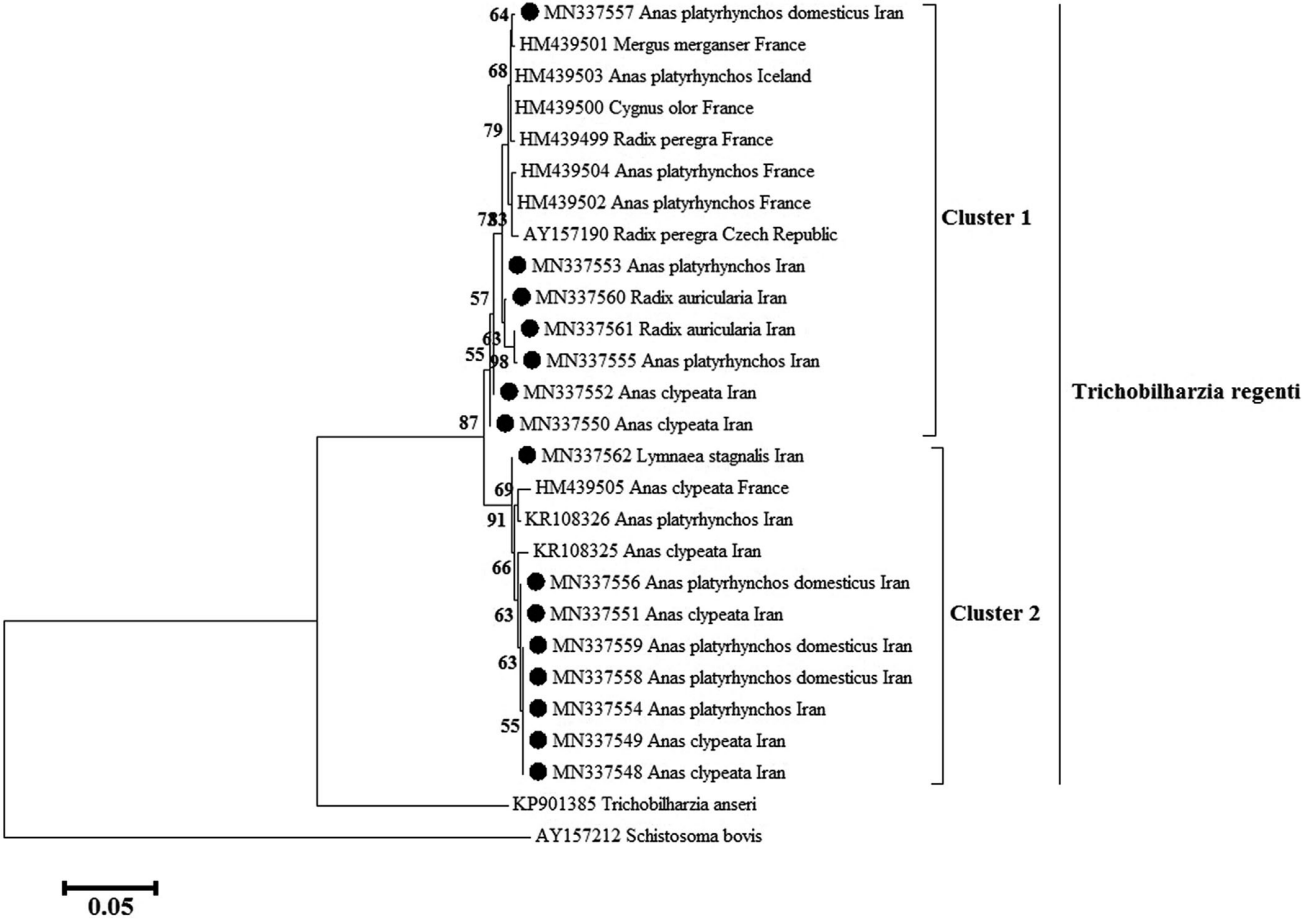
Phylogenetic relationship of the sequences of *Trichobilharzia regenti* identified in this study and known sequences previously published in GenBank as inferred by maximum‐likelihood analysis of cytochrome oxidase subunit I (COX1) sequence calculated by Hasegawa‐Kishino‐Yano (HKY+G) model. The numbers on the branches are per cent bootstrap values from 1000 replicates. Sequences of *T. regenti* detected in this study are highlighted with the black circle.

It should be noted that schistosome eggs isolated from the nasal area of 12 birds and 3 infected snails were examined for ocellate furkocercariae. These 12 birds include 4 native mallards *Anas platyrhynchos domesticus*, 3 wild mallards *A. platyrhynchos* and 5 northern shoveler *Spatula clypeata*. The infected snails were *Radix auricularia* (2 cases) and *Lymnaea stagnalis* (1 case). Regarding the COX1 gene, the phylogenetic tree of the sequences was drawn using MEGA6 software and maximum‐likelihood method, as shown in Figure 1. All schistosomes parasites identified in the present study are located in the clade related to *T. regenti*. *Trichobilharzia regenti* sequences were assigned to two clusters with a high bootstrap value (87) including cluster 1 and cluster 2. *Trichobilharzia regenti* sequences from this study clustered with those derived from several hosts from France, Czech Republic and Iceland, forming a distinct cluster (designated as cluster 1). Of the 15 obtained sequences in this study, seven belonged to cluster 1:3 sequences of *T. regenti* obtained from *Radix auricularia* (2 cases) and *Lymnaea stagnalis* (1 case) snails, 3 sequences from 1 case *Anas platyrhynchos domesticus* and 2 cases *A. platyrhynchos*, and 3 sequences from *Spatula clypeata*.

In cluster 2, eight schistosomes sequences from *Anas platyrhynchos domesticus* and wild *A. platyrhynchos, Spatula clypeata* and *Lymnaea stagnalis* grouped together with previously sequences from Iran (*Trichobilharzia cf. regenti*) and one strain from France. It is worth noting that, according to the researchers in the previous study (Fakhar et al., 2016), this cluster may represent a subtype or even a new species of *Trichobilharzia* found in the nasal area of birds. There was no geographic or host segregation observed in *T. regenti*, as COX1 sequences from this study were distributed across two clusters shown in Figure 1.

### 3.4 Results of sequencing of ITS1 gene region

**FIGURE 2:**
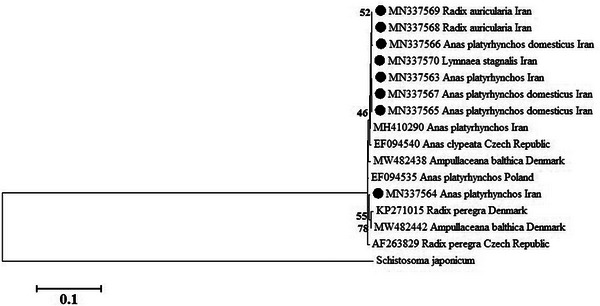
Phylogenetic relationship of the sequences of *Trichobilharzia regenti* identified in this study and known sequences previously published in GenBank as inferred by maximum‐likelihood analysis of Internal transcribed spacer subunit I (ITS) sequence calculated by Hasegawa‐Kishino‐Yano (HKY+G) model. The numbers on the branches are per cent bootstrap values from 1000 replicates. Sequences of *T. regenti* detected in this study are highlighted with the black circle.

Schistosome eggs isolated from the nasal area of 5 birds and 3 infected snails were examined for the presence of ocellate furkocercariae. The five birds included three *Anas platyrhynchos domesticus* and two *A. platyrhynchos*. In contrast to the COX1 gene region, the branches on the ITS1 ML phylogenetic tree constructed using sequences obtained from this study and retrieved from GenBank were poorly resolved. The ITS sequences did not provide enough variation to segregate the isolates into discrete clusters, including *Trichobilharzia* cf. regent and *T. regenti*. There was no observed geographic or host segregation in *T. regenti*, as COX1 and ITS1 sequences from this study were distributed across two clusters shown in Figure 2.

### 3.5 Results of haplotype network

**FIGURE 3:**
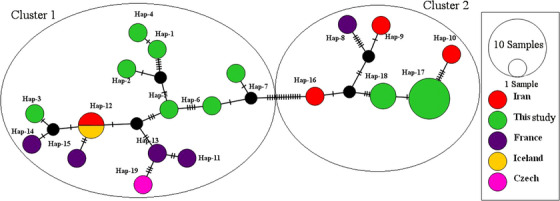
Haplotype network inferred by used haplotypes of cytochrome oxidase subunit I (COX1) estimated by Pop ART software and TCS algorithm. Each circle represents a unique haplotype and the circle size reflects frequency. The mutational steps are indicated by the short marks crossing the connection lines.

**FIGURE 4:**
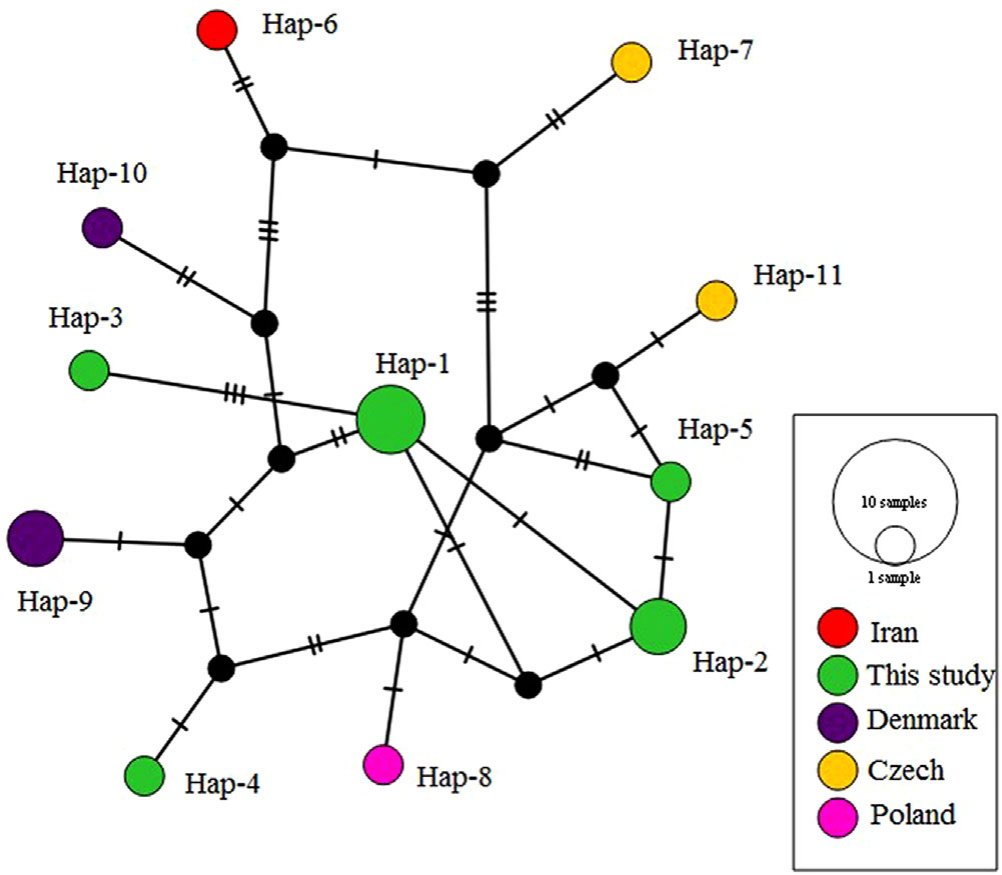
Haplotype network inferred by used haplotypes of internal transcribed spacer I (ITS1) estimated by PopART software and TCS algorithm. Each circle represents a unique haplotype and the circle size reflects frequency. The mutational steps are indicated by the short marks crossing the connection lines.

The haplotype network constructed using the TCS method included 19 haplotypes, consisting of 10 haplotypes from this study and 20 retrieved sequences from the GenBank database. These haplotypes were assigned to two clusters (Figure 3), which were consistent with those identified by Structure analysis. Two clusters, labelled as cluster 1 and cluster 2, were separated by 15 mutational steps. The haplotype network also revealed frequent sharing of haplotypes between different countries. However, similar to the phylogenetic tree constructed using the ITS1 gene, the haplotypic network of ITS1 did not show any distinct clusters (Figure 4).

## 4 DISCUSSION

This study aimed to complete the epidemiology of HCD as an endemic parasitic disease in northern Iran (Fakhar et al., 2016; Gholami et al., 2021). The first step towards achieving this goal was to investigate the final and intermediate hosts of the schistosomes to establish the parasite cycle. For the first time, the results of this paper illustrate a detailed molecular phylogenetic position of *Trichobilharzia cf. regenti* species that occur in the nasal canals of domestic ducks in northern Iran. It should be noted that previous studies have shown wild ducks infected with *Trichobilharzia cf. regenti*. Therefore, our study identified *T. cf. regenti* and *T. regenti* as the causative agents of schistosomes in the nasal area of ducks in Mazandaran Province. Similar studies conducted in European countries such as the Czech Republic, Poland, France, Belarus, and Iceland have also identified *T. regenti* as the cause of bird schistosomes in those areas (Rudolfova et al., 2002). Jouet et al. (2009) reported the *T. regenti*‐like haplotype from *Spatula clypeata*, and its snail host is also unknown. These researchers believe that this haplotype has been transferred to France through the migration of *Spatula clypeata*.

Based on previous studies in Iran, two species of *T. regenti* and *T. cf. regenti* have been identified, with confirmation through the use of molecular methods by Fakhar et al. (2016), Maleki et al. (2012) and Ashrafi et al. (2021). Maleki et al. (2012) reported the presence of *T. regenti* in *Spatula clypeata* ducks in 2012, while Fakhar et al. (2016) reported *T. cf.regenti* in *Anas clypeata* and *Anas platyrhynchos* in 2016. Earlier studies conducted in Guilan Province, also located in the western part of the southern coast of the Caspian Sea, northern Iran, revealed that domestic mallards (*A. p. domesticus*) serve as reservoir hosts for *T. regenti* and *T. franki*. These findings suggest that these parasites are not only limited to Europe but are likely endemic to Eurasia, a conclusion supported by the present study.

Also, considering the genomic similarity of half of the samples in the present study and the previous study in Mazandaran Province (Fakhar et al., 2016) with the parasite isolated from *S. clypeata* in France, it indicates that the origin of this haplotype in birds of northern Iran is Eastern Europe. Due to the migratory nature of *S. clypeata*, this haplotype may have been transmitted through the migration of this bird from Eastern Europe (Bayssade‐Dufour et al., 2006; Jouet et al., 2009).

The findings of the present study showed that the new genotype of *Trichobilharzia cf. regenti* is rotating in the study areas between migratory and domestic ducks and confirms that the establishment of the disease cycle is not dependent solely on migratory birds. Moreover, the isolation and genotyping of furkocercariae from *L. stagnalis* snails and its consistency with the newly isolated genotypes from ducks prove, for the first time in Iran, the role of *L. stagnalis* snails in the transmission of the disease in Mazandaran Province. Thus, the puzzle of the disease has been completed.

The present study is the first to have identified *Trichobilharzia* cercariae from *L. stagnalis* and *Radix auricularia* by molecular methods in Iran. The COX1 region sequence of *Trichobilharzia* furkocercariae isolated from *L. stagnalis* showed similarity to *T. cf. regenti* genotype, which is similar to most of the ducks studied. *Trichobilharzia* isolated from *Radix auricularia* also showed similarities with *T. regenti* isolated from swan and *Radix peregra* in France (Jouet et al., 2010).

Since domestic ducks do not migrate, there must be a local population of snails that can host *T. regenti* to infect them. This study reports a native cycle of this form of *Trichobilharzia* parasite in *A. p. domesticus*, *A. clypeata* and *Anas platyrhynchos* in the Mazandaran Province of Iran. The parasite has also been isolated from the host snail of *L. stagnalis* collected at Ezbaran village in Fereydoon Kenar district in the central zone of this province. Therefore, for the first time in the world, the native cycle of this form of *Trichobilharzia* parasite is reported in this region of Iran.

Among the snails collected from different parts of the province, two *Radix auricularia* snails and one *L. stagnalis* snail were infected. Studies have shown that Mazandaran Province is home to different species of Lymnaeidae snails which have the potential to serve as hosts for the parasite. In some studies in Iran, there is evidence that *R. auricularia* ( = *L. gedrosiana*) is an intermediate host of avian schistosomes (Athari et al., 2006; Farahnak & Essalat, 2003; Gohardehi et al., 2013). In addition, a study by Gohardehi et al. (2013) reported bird schistosomes in *L. stagnalis*. Despite these findings, none of the *Trichobilharzia* cercariae discovered in Iranian snails have been confirmed through molecular testing (Athari et al., 2006; Gohardehi et al., 2013). Yakhchali et al. (2016) reported two *Trichobilharzia* species (*T. franki* and *T. szidati*) from cercariae released from *R. auricularia* in northwestern Iran, which were similar to common species found in European ducks. Germany and France are among the countries where infections with nasal *Trichobilharzia* species in *R. auricularia* and *L. stagnalis* have been documented (Faltýnková & Haas, 2006; Ferte et al., 2005; Loy & Haas, 2001; Żbikowska, 2004). In general, the presence of larval stages of trematodes in *R. auricularia* and *L. stagnalis* shows the important role of these snails in transmitting the parasite to both wild waterfowl and domestic birds in the study areas. This finding is confirmed by the data collected in the study.

To date, various fragments of ribosomal and mitochondrial DNA have been used to identify phylogenetic analysis of schistosomes species. In this study, the ITS1 region was also used. However, as previously mentioned, the presence of repetitive sequences in this gene fragment can cause difficulties with sequencing and matching of fragments using two forward and reverse primers. Therefore, the use of the ITS1 region sequence for the taxonomy of *Trichobilharzia* parasites is less effective compared to the sequence of the COX1 region. To use the ITS1 region sequence for these parasites, it is best to use a pure population of the parasite and perform cloning before sequencing. In our findings, better results were obtained in determining the species of *Trichobilharzia* parasites using the COX1 region.

In conclusion, the findings of the present study suggest that the new genotype of *Trichobilharzia cf. regenti* is circulating between migratory and domestic ducks in the study areas, and confirms that the establishment of the disease cycle is not solely dependent on migratory birds. Furthermore, by isolating and genotyping furkocercariae from *L. stagnalis* and finding consistency with isolated new genotypes from ducks, this study completed the puzzle of the disease in Mazandaran Province for the first time in Iran, and demonstrated the potential role of *L. stagnalis* snails in transmitting the disease. Additionally, our study revealed that the COX1 marker has a high potential in determining the subspecies of the *Trichobilharzia* parasite; therefore, it is recommended to use this marker in future studies.

## AUTHOR CONTRIBUTIONS

MF, MK and ShG designed the study. MF and EK contributed to all parts of the study. MK and MF contributed in the analysis and interpretation of data. SD, EK, MF, MK and AB collaborated in the manuscript writing and revision. All the authors commented on the drafts and accepted the final version of the manuscript.

## CONFLICT OF INTEREST STATEMENT

The authors have no conflicts of interest to declare for this study.

## ETHICS STATEMENT

The current study was reviewed and approved by the Ethical Committee of Mazandaran University of Medical Sciences, Sari, Iran (IR.MAZ.REC.1397.1692).

### PEER REVIEW

The peer review history for this article is available at https://publons.com/publon/10.1002/vms3.1225.

## Data Availability

The author has provided the required Data Availability Statement, and if applicable, included functional and accurate links to said data therein.
